# Pulmonary Histoplasmosis, Taiwan, 1997–2024

**DOI:** 10.3201/eid3201.251091

**Published:** 2026-01

**Authors:** Ting-Wei Kao, Shang-Chen Yang, Hsiang-Wei Hu, Yu-Tsung Huang, Chin-Chung Shu, Wang-Huei Sheng

**Affiliations:** National Taiwan University Hospital, Department of Internal Medicine, National Taiwan University College of Medicine, Taipei, Taiwan (T.-W. Kao, S.-C. Yang, C-.C. Shu, W.-H. Sheng); National Taiwan University Hospital, Department of Pathology, Taipei (H.-W. Hu); National Taiwan University Hospital, Department of Laboratory Medicine, National Taiwan University College of Medicine, Taipei (Y.-T. Huang); Graduate Institute of Clinical Medicine, National Taiwan University College of Medicine, Taipei (C.-C. Shu); National Taiwan University Hospital Hsinchu Branch, Department of Internal Medicine, Hsinchu, Taiwan (W.-H. Sheng)

**Keywords:** histoplasmosis, dimorphic fungi, fungi, *Histoplasma capsulatum*, non-endemic regions, pulmonary mycosis, Taiwan

## Abstract

Pulmonary histoplasmosis has traditionally been considered geographically restricted to disease-endemic regions. Taiwan, historically nonendemic, has recently witnessed rising infections. We conducted a retrospective study by reviewing adult patients in Taiwan who had pathologically confirmed pulmonary histoplasmosis during June 1997–December 2024. We analyzed 14 cases with lung involvement. Eight case-patients were male and 6 female; mean age was 56.6 years. Of note, 11 case-patients (78.6%) had no history of travel to histoplasmosis-endemic regions; 10 (71.4%) were immunocompetent. Left upper lobe involvement was most common (n = 4 [28.6%]), with nodular lesions predominating (n = 12 [85.7%]). Most (11 [78.6%]) patients received antifungal therapy, mostly with voriconazole. Outcomes were favorable; 1 (7.1%) patient died. Two additional case-patients without lung involvement exhibited similar demographics and clinical outcomes. Case identification rate has increased since 2015. This 27-year study documents the emergence of pulmonary histoplasmosis in Taiwan, emphasizing the need for heightened clinical suspicion in nonendemic regions.

Histoplasmosis, caused by the thermally dimorphic fungus *Histoplasma capsulatum*, is a well-established disease pathogen. The clinical spectrum of histoplasmosis ranges from asymptomatic infection to life-threatening disseminated disease, depending on the extent of exposure and host immune status (*1*). In immunocompromised patients, particularly those with HIV infection or hematologic malignancies or who are receiving immunosuppressive therapy, histoplasmosis can manifest as progressive disseminated disease with high mortality rates (*2*). In immunocompetent hosts, complications rarely develop but indicate treatment once present (*3*). Common manifestations include mediastinal lymphadenitis or fibrosis and chronic pulmonary sequalae (*4*).

Traditionally, histoplasmosis has been considered a geographically restricted mycosis confined to well-defined endemic locations. The organism is found predominantly in soil enriched with bird excreta. Evolving epidemiologic reports have shown that histoplasmosis, previously confined to the endemic area of the Ohio and Mississippi River basins, can occur anywhere in the United States (*5*,*6*). Central and South America represented the other established endemic regions (*7*,*8*). Cases identified outside those regions have been predominantly attributed to travel exposure or reactivation of latent infections acquired during prior visits to endemic areas. Nevertheless, the conventional understanding of histoplasmosis as exclusively associated with endemic regions has been challenged by case reports from areas previously considered nonendemic worldwide (*9*). Those reports suggest that the environmental niche of *H. capsulatum* fungus may be more extensive than previously recognized and so might have changed environmental conditions, intensified global travel, and improved diagnostic capabilities. Furthermore, the organism’s capability to establish environmental reservoirs in new geographic locations challenges traditional disease transmission patterns and epidemiologic surveillance approaches. Enabled by the ecologic preference for nitrogen-rich soil, especially soil contaminated with bird and bat guano, and the association with avian and chiropteran hosts that can disperse the fungus across regions (*10*), *Histoplasma capsulatum* has a documented capability to establish environmental reservoirs in new geographic locations. Molecular surveillance and ecologic niche modeling have reported that *Histoplasma* spp. can persist in both traditional endemic and newly recognized areas (*11*).

Taiwan, situated in the Western Pacific region, has traditionally been considered nonendemic for histoplasmosis based on limited historical documentation and the absence of systematic surveillance reports. A total of 17 cases in Taiwan during 1977–2022 were identified by literature review (*12*); those reports did not detail possible exposure by occupation, condition and characteristics of case-patients’ lungs, or outcomes. Still, those cases have delineated an evolving epidemiologic landscape that warrants comprehensive investigation (*13*). The increasing recognition of histoplasmosis cases in Taiwan, in conjunction with the heterogeneous clinical manifestations and variable host–pathogen interactions, necessitates detailed characterization of disease burden and clinical characteristics. Therefore, our study aimed to elucidate the demographic, clinical, radiologic, and therapeutic characteristics of pulmonary histoplasmosis in Taiwan over a 27-year period.

We observed the ethical principles of Declaration of Helsinki in designing and conducting this study. The research ethics committee of National Taiwan University Hospital approved the study (no. 202506155RINB); informed consent was waived because of the retrospective nature of the study.

## Materials and Methods

### Study Design and Participants 

**Figure 1:**
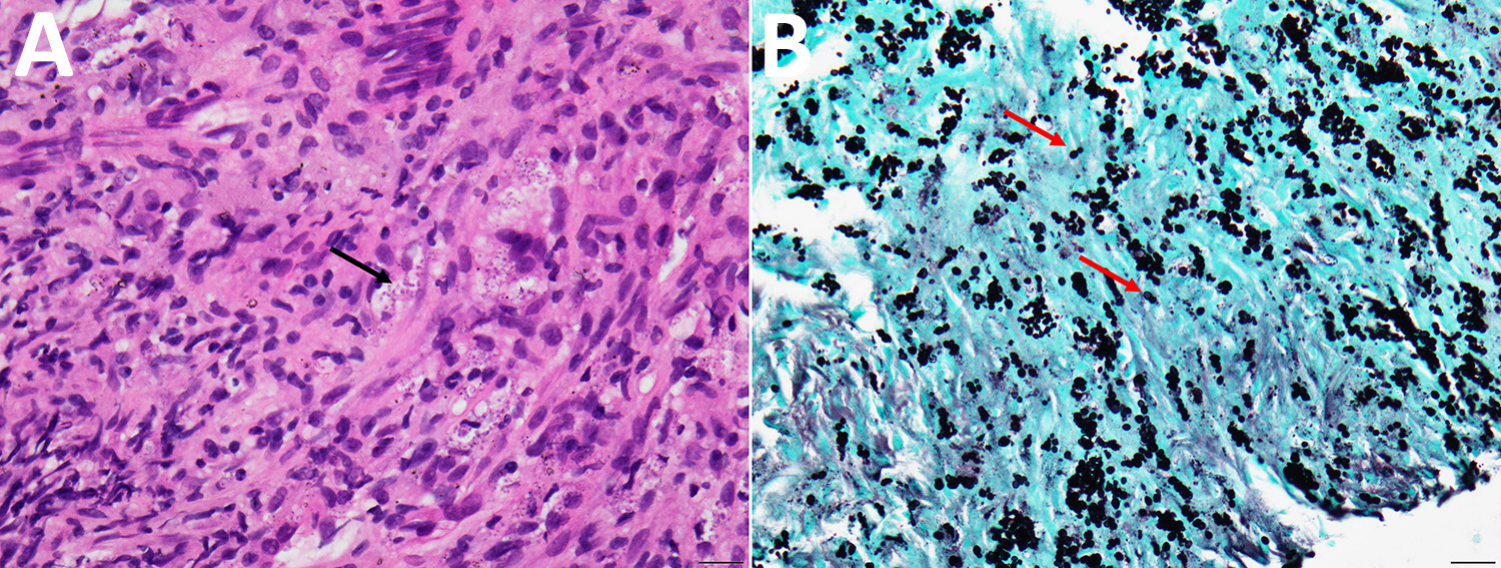
Representative histopathologic findings of pulmonary histoplasmosis in Taiwan, 1997–2024. A) Hematoxylin and eosin stain delineates numerous tiny ovoid yeasts within macrophages; occasional narrow-based budding indicated (black arrow). B) Grocott's methenamine silver stain shows yeasts with narrow-based budding (red arrows). Scale bars represent 20 µm.

We conducted a retrospective observational study at National Taiwan University Hospital, a 2,600-bed tertiary medical center in Taipei, Taiwan. We designed the study protocol to include all cases of histoplasmosis diagnosed during June 1997–December 2024. We systematically identified participants through an exhaustive search of the electronic medical record database using diagnosis coding for histoplasmosis from the International Classification of Diseases, 10th Revision. We defined the inclusion criteria as adult patients (>20 years of age) with pathologically confirmed histoplasmosis. All tissue specimens underwent standardized processing protocols including hematoxylin and eosin staining, periodic acid-Schiff staining, and Gomori methenamine silver staining for organism detection and morphological characterization. Experienced pathologists confirmed histoplasmosis diagnosis on the basis of concomitant presence of characteristic intracellular yeast forms of *H. capsulatum* in tissue specimens, 2–4 µm in size, with narrow-based budding, and in the absence of transverse septum (Figure 1). In addition, the primary treating physician determined pulmonary involvement on the basis of clinical assessment, radiologic evidence, and microbiologic findings from respiratory tract specimens or lung tissue.

### Data Extraction

For each identified case, we systematically retrieved demographic information including age at diagnosis, sex, body mass index, smoking history, occupation, pet ownership, residential distribution, and detailed travel history within 6 months preceding diagnosis. We used Centers for Disease Control and Prevention definitions for endemicity for travel destination (*14*). We also documented clinical data including cardinal manifestations of pulmonary histoplasmosis (fever, dyspnea, cough, and bodyweight loss), underlying conditions, immunosuppression factors, and antifungal medication. We defined immunosuppression as those who received immunosuppressive therapy. We systematically reviewed chest imaging studies to characterize the location, extent, and pattern of pulmonary involvement. We classified lesions by morphologic appearance and anatomic distribution by pulmonary lobe.

### Statistical Analysis

We performed statistical analyses using SPSS Statistics 19.0 software (https://www.ibm.com) and compiled descriptive statistics as mean +SD for continuous variables and frequencies with percentages for categorical variables. Because the sample size was relatively small for this rare disease, we compared groups using nonparametric tests: Mann-Whitney U test for continuous variables and Fisher exact test for binary categorical variables. We defined statistical significance as a 2-sided p value <0.05.

## Results

A total of 14 persons with pathologically confirmed histoplasmosis and lung involvement comprised the primary cohort for analysis. We identified 2 additional patients without lung involvement for comparison (Table 1). The population was predominantly male (8 [57.1%] vs. 6 [42.9%] female); mean age was 56.6 years. Most patients were never smokers (12 [85.7%]); the remaining 2 had a history of former smoking. Regarding geographic distribution, 10 (71.4%) patients resided in northern Taiwan, and 4 (28.6%) patients were from the middle Taiwan regions. Of note, occupational exposure to dust or soil environments was present in 3 (21.4%) patients; 1 (7.1%) was a pet owner. Three (21.4%) patients had a history of travel to recognized disease-endemic areas.

**Table 1:** Characteristics of case-patients in study of pulmonary histoplasmosis in Taiwan, 1997–2024*

Case no.	Age, y/ sex	Date	Travel history	Occupation	Pet	Residence region	Involved site (details)	Immunocompromised factor	Antifungal agent
With lung involvement									
1	21/F	2001 Apr	USA (Illinois)†	Teacher	None	North	Lung (nodule)	Immunocompetent	None
2‡	86/M	2003 Feb	Indonesia,† Thailand,† Saudi Arabia, China (Shandong)	Excavator driver	None	North	Lung (nodule)	Addison’s disease	Amphotericin B
3	71/M	2014 Aug	India,† Guatemala,† USA (California)	Electronics	None	North	Lung (nodule)	Immunocompetent	None
4	20/F	2016 Jul	None	Student	None	North	Lung (nodule)	Immunocompetent	Voriconazole
5	74/F	2016 Jul	None	Housewife	None	North	Lung (nodule)	Immunocompetent	Posaconazole
6	36/M	2019 Aug	None	Office worker	None	North	Lung (nodule)	Immunocompetent	Voriconazole
7	32/M	2019 Dec	None	Unknown	None	Middle	Lung (nodule)	Immunocompetent	Posaconazole
8	66/M	2020 May	None	Unknown	None	North	Disseminated (involving skin, lymph node, and GI tract)	Kidney transplant	Voriconazole
9	54/M	Mar 2021	None	Electronic engineering	None	North	Lung (nodule)	Immunocompetent	Itraconazole
10	53/M	Oct 2021	None	Cement worker	None	Middle	Lung (nodule)	Immunocompetent	None
11	83/F	Dec 2021	None	None	None	North	Lung (consolidation)	Rheumatoid arthritis	Voriconazole
12	63/F	Sep 2023	None	Housewife	None	Middle	Lung (nodule)	Immunocompetent	Fluconazole
13	60/F	2023 Sep	None	Indoor decoration	Duck	North	Lung (ill-defined opacity), bone marrow	Myelodysplasic syndrome	Ambisome
14§	74/M	2024 Sep	None	Cement worker	None	Middle	Lung (nodule)	Immunocompetent	Voriconazole
Without lung involvement									
15	27/M	1997 Jun	Malaysia,† Thailand,† Singapore†	Unknown	None	North	Disseminated (involving lung, bone marrow, GI tract, and lymph node)	HIV	Amphotericin B
16	78/M	2003 Oct	None	Farmer	None	East	Mediastinal lymph node	None	Amphotericin B

*GI, gastrointestinal. †These areas likely to be hyperendemic, cases likely to occur regularly, and locally acquired cases to be reported. ‡Patient died of respiratory failure. §Patient survived with Evans syndrome.

Clinical manifestations varied across the cohort. When assessed individually, each cardinal symptom was present in 21.4%–35.7% of the study population. However, up to 64.3% of the patients manifested a constellation of these symptoms. Underlying conditions were notable within the study population. Two (14.3%) patients had a history of tuberculosis; 1 experienced remote infection, and 1 had a concurrent case of pulmonary histoplasmosis. Four (28.6%) patients were receiving immunosuppressive therapies: 2 patients for autoimmune diseases, 1 patient for active hematologic malignancy, and 1 patient for solid organ transplantation. Diagnostic confirmation of histoplasmosis was achieved primarily through histopathologic examination, whereas microbiologic confirmation through positive fungal culture was achieved in 2 (14.3%) patients.

**Figure 2:**
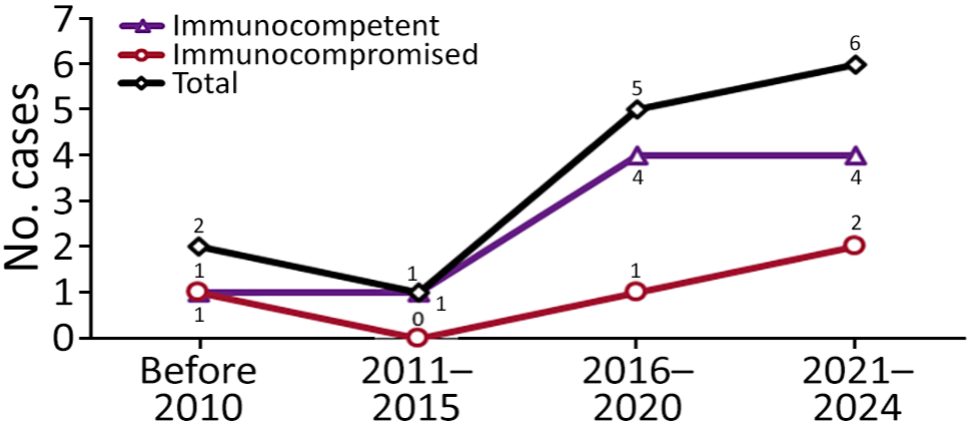
Epidemiologic trend of pulmonary histoplasmosis in Taiwan, stratified by host immunity, 1997–2024. Numbers above data points indicate numbers of cases.

**Figure 3:**
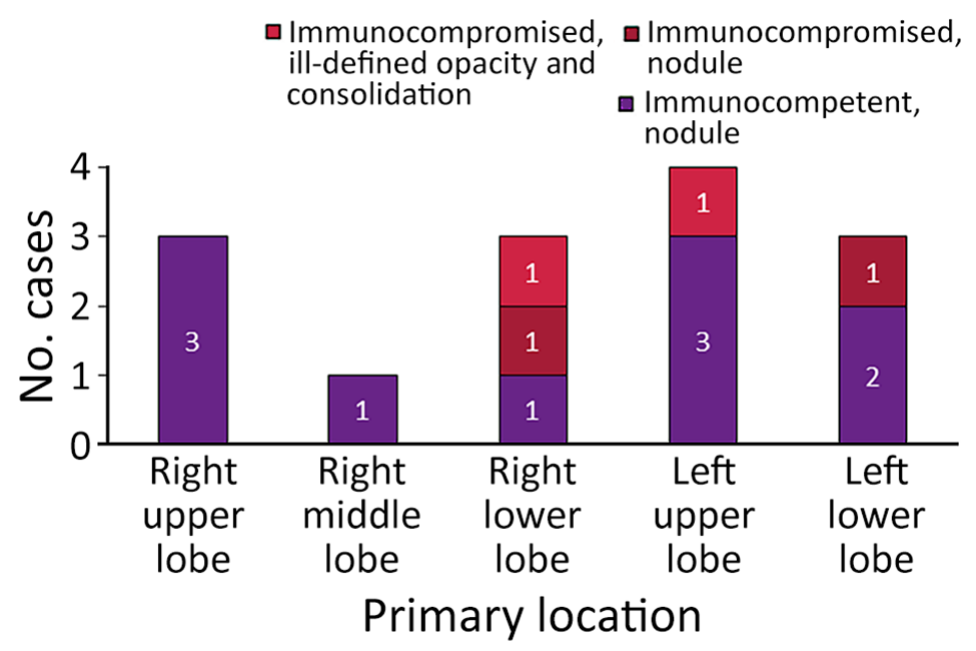
Locations and characteristics of primary lung lesions in patients with pulmonary histoplasmosis in Taiwan, 1997–2024.

**Figure 4:**
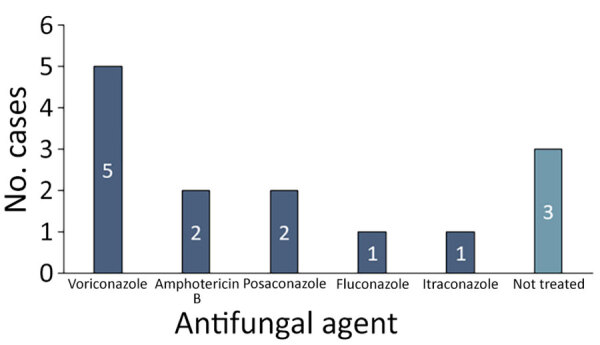
Distribution of antifungal agents used in cases of pulmonary histoplasmosis in Taiwan, 1997–2024.

We observed temporal distribution of cases during 2016–2024 (Figure 2). Throughout the study, the number of immunocompetent patients consistently exceeded their immunocompromised counterparts. We noted anatomic involvement in radiographic images consistent with typical manifestations of pulmonary histoplasmosis described in endemic regions (Figure 3). The predominant pattern was nodular lesions (12 [85.7%] patients); the 2 exceptions appeared as consolidation and ill-defined opacity. Most (11 [78.6%]) patients received appropriate antifungal treatment (Figure 4). Voriconazole was the most commonly administered therapeutic agent, followed by amphotericin B, posaconazole, fluconazole, and itraconazole.

Comparative analysis by host immunity revealed no statistically significant differences in symptom presentation, underlying conditions, absolute neutrophil count, and C-reactive protein level between the immunocompetent and immunocompromised groups. However, immunosuppressed patients were numerically but not significantly older (73.8 vs. 49.8 years of age; p = 0.07). In addition, the elderly patients demonstrated a higher tendency to exhibit cardinal symptoms (83.3% vs. 50.0%; p = 0.30). Conversely, younger patients manifested slightly lower absolute neutrophil counts (3.2 vs. 5.3 k/μL; p = 0.15).

The overall prognosis was favorable. One patient (patient 2), who was immunosuppressed from steroid use for Addison’s disease, eventually experienced severe pneumonia with hypoxemic respiratory failure despite treatment with amphotericin B (1 mg/kg/d) and died in the hospital (*15*). Patient 14 experienced consequent immune system dysregulation by manifesting idiopathic thrombocytopenic purpura and autoimmune hemolytic anemic (Evans syndrome); that patient survived till the study endpoint (*16*).

We identified 2 patients during the study period who had histoplasmosis without thoracic involvement; they experienced multiorgan involvement including the gastrointestinal tract, skin, colon, lymph nodes, and bone marrow. One patient had concurrent HIV infection with Kaposi sarcoma; the other was considered immunocompetent. Comparative analysis revealed no significant demographic or clinical parameter differences between pulmonary and nonpulmonary cases (Tables 2,3,4).

**Table 2:** Key parameters in study of pulmonary histoplasmosis by host immunity status, Taiwan, 1997–2024*

Characteristic	All, N = 14	Immunocompetent, n = 10	Immunocompromised, n = 4	p value
Mean age, y, + SD	56.6 + 21.8	49.8 + 21.2	73.8 ± 12.7	0.07
Sex				
M	8 (57.1)	6 (60.0)	2 (50.0)	1.00
F	6 (42.9)	4 (40.0)	2 (50.0)	
Any cardinal symptoms	9 (64.3)	5 (50.0)	4 (100.0)	0.22
Tuberculosis	2 (14.3)	1 (10.0)	1 (25.0)	0.49
HIV	0	0	0	NA
Travel history	3 (21.4)	2 (20.0)	1 (25.0)	1.00
Immunosuppression	4 (28.6)			
ANC + SD	4.1 + 2.1	4.2 + 1.7	3.9 + 3.4	1.00
CRP + SD	4.2 + 3.8	3.1 + 4.0	5.2 + 4.2	0.40
In-hospital death	1 (7.1)	0	1 (25.0)	0.29
Complication	2 (14.3)	1 (10.0)	1 (25.0)	0.51

*Values are no. (%) except as indicated. ANC, absolute neutrophil count; CRP, C-reactive protein; NA, not applicable.

**Table 3:** Key parameters in study of pulmonary histoplasmosis, by lung involvement, Taiwan, 1997–2024*

Characteristic	Yes, n = 14	No, n = 2†	p value
Mean age, y, + SD	56.6 + 21.8	52.5 + 25.5	1.00
Sex			
M	8 (57.1)	2 (100)	0.26
F	6 (42.9)	0 (0)	
Any cardinal symptoms	9 (64.3)	2 (100)	0.32
Tuberculosis	2 (14.3)	0	0.58
HIV	0	1 (50.0)	**0.008**
Travel history	3 (21.4)	1 (50.0)	0.40
Immunosuppression	4 (28.6)	1 (50.0)	0.55
ANC + SD	4.1 + 2.1	NA	NA
CRP + SD	4.2 + 3.8	NA	NA
In-hospital death	1 (7.1)	0	0.71
Complication	2 (14.3)	0	0.58

*Values are no. (%) except as indicated. Values in bold text are statistically significant. ANC, absolute neutrophil count; CRP, C-reactive protein; NA, not applicable. †These 2 additional cases were used as controls.

**Table 4:** Comparisons of key parameters in study of pulmonary histoplasmosis by disease manifestation in lungs, Taiwan, 1997–2024*

Characteristic	All, N = 14	Nodule, n = 12	Other, n = 2†	p value
Mean age, y, + SD	56.6 + 21.8	54.2 + 22.1	71.5 + 16.3	0.36
Sex				
M	8 (57.1)	8 (66.7)	0	0.09
F	6 (42.9)	4 (33.3)	2 (100)	
Any cardinal symptoms	9 (64.3)	7 (58.3)	2 (100)	0.27
Tuberculosis	2 (14.3)	2 (16.7)	0	0.55
HIV	0	0	0	NA
Travel history	3 (21.4)	3 (25.0)	0	0.44
Immunosuppression	4 (28.6)	2 (16.7)	2 (100)	**0.02**
ANC + SD	4.1 + 2.1	4.2 + 1.6	3.5 + 4.7	0.83
CRP + SD	4.2 + 3.8	4.7 + 4.6	3.0 + 2.3	1.00
In-hospital death	1 (7.1)	1 (8.3)	0	0.68
Complication	2 (14.3)	2 (16.7)	0	0.55

*Values are no. (%) except as indicated. Values in bold text are statistically significant. ANC, absolute neutrophil count; CRP, C-reactive protein; NA, not applicable. †These 2 additional cases were used as controls.

## Discussion

This comprehensive, longitudinal analysis of histoplasmosis cases in Taiwan over a 27-year period fundamentally challenges the established paradigm that histoplasmosis remains confined to traditionally recognized endemic geographic regions. Our findings demonstrate the emergence of histoplasmosis in Taiwan and underscore the importance of considering a histoplasmosis diagnosis even in geographic locations previously classified as nonendemic or in immunocompetent patients.

Our results extend previous observations documenting 17 histoplasmosis cases from Taiwan over 4 decades (*12*). Local *Talaromyces marneffei*, another dimorphism fungus with similar morphology, has been identified in Taiwan; *H. capsulatum* can be distinguished histologically by its pathognomonic feature of the central septum (*17*). The marked increase in diagnosed histoplasmosis cases since 2016 exemplifies enhanced case recognition, a likely result of improved diagnostic modalities (*18*). The absence of culture or molecular typing can prevent definite diagnosis, as reflected in therapeutics. Even though itraconazole had been endorsed as standard treatment (*19*), only 6% of the patients in our cohort were treated with it. Voriconazole was the most frequently prescribed alternative instead, perhaps because of its lower adverse effect on gastrointestinal tract and liver function, as well as its broader antifungal spectrum when diagnosis remains indefinite.

A notable finding of our study is the limited travel history among affected patients. National health insurance data indicated a local incidence of histoplasmosis at 0.24/100,000 population annually (*20*); our study suggested that local acquisition of histoplasmosis in Taiwan may be more common than previously recognized. Whether those cases can be attributed to indigenous transmission or imported disease remained unknown; however, our study provides more definitive evidence for local acquisition by demonstrating the high proportion of patients without relevant travel history.

We hypothesized that the environmental niche of *H. capsulatum* is geographically more widespread than previously considered (*21*). Several factors contributed to fungal growth and spore production in previously nonendemic areas, including climate change, urbanization, construction activities, soil transportation, and changes in bird population distribution. Climate change creates favorable environmental conditions for *H. capsulatum* fungus survival and proliferation in regions previously considered unsuitable. The increasing urbanization and construction activities disturbed soil containing dormant fungal spores, resulting in aerosolization and subsequent human exposure. In addition, global trade and transportation of soil materials, agricultural products, and construction materials likely enable the introduction and establishment of *H. capsulatum* in novel geographic locations. Approximately 30% of cement clinker used for construction in Taiwan is imported, which might provide a route of transmission for *H. capsulatum* and subsequently serve as an exposure source.

The emergence of histoplasmosis in immunocompetent persons represents another concerning epidemiologic trend. Previous studies have reported cases of immunocompetent hosts with disseminated histoplasmosis, although most had obvious exposure through construction work (*22*). In our series, not all cases could be identified with specific exposure sources either through occupation or travel history. A similar case report from South Korea also described a patient with pulmonary histoplasmosis who was neither immunocompromised nor had travel history (*23*). Although immunosuppression has been well established as an important predisposing factor for severe histoplasmosis, our study demonstrates that most patients were immunocompetent at the time of diagnosis. In addition to heterogeneous clinical manifestations observed in our cohort, those cases suggested that traditional risk stratification approaches might need revision and that environmental factors or genetic susceptibility play pivotal roles. Genetic polymorphisms in immune response genes may influence individual susceptibility to histoplasmosis infection and disease severity (*24*). Environmental factors, such as the intensity and duration of exposure, coexisting respiratory conditions, or concurrent infections, could also modulate disease manifestation in immunocompetent hosts. The occurrence of disseminated disease in the additional cases we describe exemplifies the potential severity of histoplasmosis even in presumably low-risk populations. The development of Evans syndrome in 1 patient further demonstrates the potential for complex immunological complications after histoplasmosis infection. Despite those complications, the favorable outcomes achieved with appropriate antifungal therapy demonstrate the efficacies of early recognition and treatment.

Of radiology results, nodular lesions remained the most prevalent pattern we observed at >80%, which closely matches previous reports (*25*). However, some studies have described additional specific patterns including cavitary lesions (*26*), halo (*27*) or reverse halo (*28*) signs, and spindle cell lesions (*29*). Because of those imaging findings, pulmonary histoplasmosis can be easily confused with primary lung malignancy or metastatic lesions, thereby mandating tissue-based diagnostic procedures. The exceptionally low yield of fungal cultures in our study emphasizes the inherent diagnostic difficulties associated with histoplasmosis; histopathologic examination remained the primary diagnostic modality. The integration of molecular diagnostic methodologies, including PCR-based assays, monoclonal antibody–based assays (*30*), and next-generation sequencing approaches (*31*), could enhance detection rates and support pathological findings. Those advanced diagnostic modalities could be particularly valuable in nonendemic settings where clinical suspicion may be lower and traditional diagnostic methods may be less reliable. Moreover, antigen detection assays, particularly urinary *Histoplasma* antigen testing, have shown promise in endemic areas to enable diagnosis in nonendemic regions, especially for disseminated cases.

Our findings underscore the critical need for heightened clinical suspicion of histoplasmosis even in previously considered nonendemic settings, particularly given the varied manifestations observed in both immunocompromised and immunocompetent patients. Future research should prioritize systematic environmental reservoir identification, genetic determinants of host susceptibility, and optimization of diagnostic approaches specific to nonendemic settings. Previous studies have applied PCR to identify local acquisition in wildlife and environment samples (*32*,*33*); we expect the molecular approach to demonstrate that epidemiology of infectious disease evolves in response to changing environmental and demographic factors, emphasizing the need for adaptive surveillance and clinical preparedness strategies.

A limitation of this study is that its retrospective nature inherently limited data completeness and potentially introduced selection bias toward more severe patients requiring invasive investigation. Potential study participants were identified by International Classification of Diseases coding and confirmed as having histoplasmosis after pathology review; most lacked positive culture of *H. capsulatum*, which would provide a definite diagnosis. A second limitation is that we documented only recent travel history in our study. Reactivation of *H. capsulatum* that was acquired from remote travel cannot be totally excluded. Third, the small sample size confined statistical power for subgroup analyses and obscured subtle but clinically important differences between different patient phenotypes; we acknowledge that the small sample reflected the rarity of the disease. Fourth, we identified the patients in our study relying on histopathologic diagnosis; we lacked culture data for validation. We might have underestimated genuine incidence of histoplasmosis because mild or asymptomatic case records might not indicate tissue sampling. Finally, we did not address the identification of local fungal reservoirs and transmission sources.

In conclusion, this 27-year case series provides evidence for the emergence of histoplasmosis in Taiwan, potentially challenging traditional geographic boundaries of such infections. The high proportion of patients without travel history to endemic regions is suggestive of local acquisition and possible *H. capsulatum* reservoirs in the environment. The predominance of immunocompetent hosts among our cases indicates evolving epidemiologic patterns that require further clinical recognition and risk assessment strategies. We recommend high clinical suspicion for histoplasmosis in patients with compatible clinical and radiologic findings, even in the absence of travel history or obvious immunocompromising conditions.
